# AI-Assisted Angoff Standard Setting for Multiple-Choice Examinations in Medical Education: Comparative Study

**DOI:** 10.2196/98766

**Published:** 2026-09-09

**Authors:** Salah Eldin Kassab, Mariam Shadan, Arina Ziganshina, Shifan Khanday, Hossam Hamdy

**Affiliations:** 1 Department of Biomedical Sciences, College of Medicine, Dubai Medical University, Al Muhaisnah 1, Al Mizhar, Dubai, United Arab Emirates, 971 505835469; 2 Department of Physiology, Faculty of Medicine, Suez Canal University, Ismailia, Egypt; 3 Department of Clinical Sciences, College of Medicine, Dubai Medical University, Dubai, United Arab Emirates

**Keywords:** large language models, standard setting, modified Angoff method, medical education, multiple-choice examinations, artificial intelligence, AI

## Abstract

**Background:**

Standard setting is essential for a defensible assessment in medical education. The modified Angoff method requires several expert judges, and determining the minimally competent candidate is cognitively challenging. However, empirical evidence on the role of AI in standard setting is unclear.

**Objective:**

This study aimed to examine the role of large language models (LLMs) in the modified Angoff standard setting method for multiple-choice questions in a basic medical science examination compared with faculty judges.

**Methods:**

This study was conducted in year 2 of the MD program in the United Arab Emirates. Ten faculty judges and 5 LLMs (GPT-5.2, Grok, DeepSeek, MedGemma, and Claude 4.5 Sonnet) determined the Angoff cutoff scores for a summative examination (120 multiple-choice questions). Standardized prompts were used for the LLMs to mimic the same information provided for faculty judges. Generalizability (*G*) theory analyses were performed using a fully crossed item × rater design to estimate variance components, *G* and Φ coefficients, decision study, and root mean squared error (RMSE) of the Angoff cutoff scores.

**Results:**

Faculty-generated Angoff estimates (mean 69.13, SD 10.92) were comparable to LLM-generated estimates (mean 68.94, SD 12.39). A 2-tailed paired-sample *t* test revealed no statistically significant difference between the 2 groups (95% CI −2.21 to 2.91; *t*_119_=0.27; *P*=.79). Generalizability theory analysis demonstrated moderate reliability for the faculty panel (*G* coefficient=0.738; Φ coefficient=0.692). Despite comprising only 5 LLMs, the LLM panel demonstrated higher reliability (*G* coefficient=0.823; Φ coefficient=0.815), lower RMSE (1.28 vs 2.33), higher item-related variance (46.85% vs 18.36%), and lower rater-related variance (2.64% vs 16.40%) than the faculty panel. Pass rates were similar using LLM- and faculty-derived cutoff scores (52/73, 71.2%). LLMs differed in their minimally competent candidate conceptualization and approaches to determining item-level percentages of correct responses. Furthermore, the correlations between Angoff estimates and item-related *P* values were larger in LLMs than in faculty judges (*r*=0.552 vs 0.437).

**Conclusions:**

In this single-institution study, the evaluated LLMs generated modified Angoff estimates that were broadly comparable to those of faculty judges. Generalizability theory analyses demonstrated higher *G* and Φ coefficients, lower rater-related variance, and lower RMSE for the evaluated LLM outputs under standardized prompting conditions, indicating greater consistency in the generated estimates. Application of the resulting cutoff scores produced pass and fail rates similar to those derived from faculty judges. These findings suggest the future application of LLMs as decision assistance tools for modified Angoff standard setting while maintaining expert human oversight.

## Introduction

### Background

Standard setting is an essential quality measure for student assessment, ensuring that decisions about examination scores reflect meaningful levels of student competence [1]. Unlike using fixed cutoff scores, standard setting provides a flexible approach to align pass and fail decisions with examination difficulty. This process includes a systematic integration of expert judgment to determine the cutoff score that distinguishes pass from fail or competent from noncompetent candidates. There is no “gold standard” for determining the cutoff score for the pass and fail decisions. However, the credibility of these decisions depends on the rigor and defensibility of and well-supported evidence on the process [2]. Furthermore, the generated cutoff score depends on the method of standard setting used and the panel of expert judges [3,4].

The Angoff method is widely used for determining cutoff scores based on expert judgment of minimally competent candidate (MCC) performance [5-8]. This method has the advantage of being criterion referenced, which ensures alignment with established professional competence frameworks [9]. In contrast, norm-referenced methods carry the risk of erroneous promotion of incompetent candidates and the inappropriate failure of competent candidates. Multiple variants of the Angoff method have been reported in the health professions education literature, among which the modified Angoff method with reality check achieved the highest reliability, representing the most defensible and consistent procedure [9,10]. However, this process is often time-consuming and resource intensive [5]. In addition, conceptualizing the MCCs represents a cognitive burden on the expert judges [11]. Therefore, extensive training of the expert panelists is an essential requirement to reduce the misconceptions among judges and subjective variability of the cutoff scores [12].

### Prior Work

With the increasing capabilities of large language models (LLMs), there is growing interest in exploring how AI can assist in student assessment [13-15]. However, there is very limited peer-reviewed research in medical education on the use of AI for evaluating Angoff standard setting in a manner comparable to that of human expert panels [14]. The integration of AI into standard setting could be conceptualized through the substitution, augmentation, modification, and redefinition (SAMR) theoretical framework [16], which describes 4 levels of integrating technology into educational practices [16]. At the substitution level, AI may substitute the faculty role by conceptualizing the MCC and generating the Angoff estimates. At the augmentation level, AI can work as a collaborative partner to improve the efficiency of the process through rapid analysis and feedback that supports faculty judgment while maintaining human oversight [17]. At the modification and redefinition levels, AI can modify the process by synthesizing judgments and potentially redefine standard setting practice to refine Angoff estimates. However, the SAMR model should not be viewed as a rigid hierarchy but as a flexible framework to guide the meaningful integration of AI into the standard setting context.

### Study Objectives

This study examined how LLMs can support the modified Angoff standard setting process for multiple-choice question (MCQ) examinations using the SAMR framework. Specifically, we assessed whether LLMs can replicate faculty judgment (substitution) or enhance the uniformity and exactness of standard setting (augmentation). This study addressed three questions: (1) Are there significant differences between Angoff estimates from faculty judges and LLMs? (2) How do different LLMs conceptualize the MCC and estimate item-level probabilities? (3) What evidence supports the reliability and credibility of Angoff estimates from faculty vs LLMs?

## Methods

### Ethical Considerations

The Institutional Research Ethics Committee of Dubai Medical University approved this study (approval number DMU/IRB/OEJW9A/2026/30). Participation of faculty judges was voluntary, and we obtained informed consent from each participant prior to their involvement in the study. Individuals were informed that they may withdraw at any stage without penalty. Anonymity and confidentiality were maintained by assigning unique study codes to all expert contributors and removing any identifying information from records. All study data were securely stored in password-protected files accessible only to the study authors. Similarly, all student examination data were anonymized prior to analysis, and no individual-level student identifiers were accessible to either the human judges or the LLMs. The dataset provided to the LLMs included cumulative grade point averages (GPAs) together with study-specific anonymized identification codes used solely to distinguish individual records during analysis. Institutional student identification numbers and all other personally identifiable information were removed before the data were uploaded in the PC of the first author (SEK). The anonymized study codes could not be linked to individual students by the LLMs or any third-party service.

### Study Context

This study was conducted in year 2 of the preclinical phase of the MD program at a medical school in the United Arab Emirates. The 6-year MD program encompasses 226 credit hours and consists of 3 phases, each with a duration of 2 years. Phase 1 of the program consists of foundational courses in basic medical sciences and general education. Phase 2 constitutes integrated, system-based courses with early clinical exposure and research training. Phase 3 consists of hospital-based clerkships in clinical specialties and subspecialties.

### Study Design and Sampling

This study was designed to compare the generated cutoff scores using the modified Angoff method by faculty judges (n=10) vs 5 LLMs (generative pretrained transformer [OpenAI], Grok [SpaceXAI LLC], DeepSeek, MedGemma [Google DeepMind], and Claude [Anthropic]). This study was conducted on a summative final examination of basic medical sciences that consisted of A-type (single best response) MCQs with 4 options. The content of the examination was related to biochemistry, molecular biology, genetics, and fundamentals of pharmacology. Test items were constructed by subject matter experts followed by peer review and then validated by the college assessment committee members to ensure content validity and correct any technical flaws in the design.

### Standard Setting Methods

#### Conducting the Modified Angoff Procedure by Faculty Judges

Prior to the standard setting exercise, faculty judges (n=10) participated in a structured training program that consisted of 2 sessions of 1 hour each delivered by the first author (SEK). The judges were selected from a pool of basic sciences faculty who were familiar with the curriculum, cohort of students, and student assessment system and had at least 2 years of experience teaching this curriculum. The 10-judge panel consisted of 4 (40%) biochemistry faculty members, 2 (20%) molecular and cell biology faculty members, 2 (20%) pharmacology faculty members, and 2 (20%) lecturers in biomedical sciences.

The sessions focused on the definition of standard setting, the purpose of its implementation, and the steps of determining the cutoff scores using the modified Angoff method. An essential aspect of the training was how to conceptualize the MCC. The following definition was adopted for the MCC: “A student who studies an average amount of time, possesses knowledge which is sufficient for borderline pass, but struggles to achieve scores above the cut-off threshold” [18]. The sessions ended with hands-on training on 5 MCQs outside the examination content by the faculty judges and elaboration on the individual differences between the generated Angoff estimates. At the end of the training sessions, faculty judges were provided with a Microsoft Excel sheet on the previous cumulative GPAs of the cohort of students. This “reality check” process aimed to reduce the variability between the judges in conceptualizing the MCC group of students. Faculty judges were then asked to independently estimate how much the minimally competent group of students was expected to score on each test item. The 10 faculty judges independently rated all 120 examination items. Correct answers to the test items were provided to the judges. However, there was no group discussion or establishment of consensus for each test item. The average scores from each faculty judge were calculated, and the final cutoff score represented the mean estimates of the 10 judges.

#### Prompting Process for Conducting the Angoff Procedure by the LLMs

The specific versions of the LLMs evaluated were GPT-5.4, Grok 4.20, DeepSeek-V3.2, Claude 4.5 Sonnet, and MedGemma 1.5, each accessed via their respective standard web-based chat interfaces or via Google’s Gemini Enterprise Agent Platform Model Garden in the case of the latter. All models were used with each platform’s default generation settings and were accessed between December 2025 and March 2026. A standardized prompting protocol for LLMs was designed to ensure the reproducibility of the study findings (Multimedia Appendix 1). The prompts were designed to match the same information provided to the faculty judges during the training session. Specifically, each LLM was fed the following information: (1) current GPA of the cohort under study; (2) content-validated MCQs with the correct answers; and (3) a Microsoft PowerPoint presentation describing the process of standard setting, rationale for its use, modified Angoff method, and concept of the MCC. The prompt to each LLM was to assume the role of a faculty expert who was participating in the modified Angoff method and identify the expected percentage of MCC students who scored each item correctly. Following generation of the initial output, each LLM was prompted to articulate its definition of the MCC and explain the rationale for the estimated percentage score for the items. The wording of the prompts, sequence of the uploaded information, and instructions were used consistently across all 5 LLMs with no change or modification. Each LLM was blinded to the scores of the faculty judges and the outputs of the other models. The overall LLM cutoff score was generated from the average scores of the 5 models.

#### Cohen Method for Standard Setting

We also compared the application of the modified Angoff method with the method by Cohen-Schotanus and van der Vleuten, which is a norm-referenced approach to standard setting [19,20]. The cutoff score was determined using the following formula:

Cutoff score = *cN* + 0.60 (N* – *cN*)

In this equation, *c* is the proportion of correct responses expected by chance (0.25 for a 4-option multiple-choice examination), *N* is the total number of examination items (120 in the present study), and N* is the raw examination score of the student at the 95th percentile of the score distribution.

### Statistical Analysis

Descriptive statistics were calculated for all cutoff scores, including means and SDs. To account for measurement error in observed examination scores, the estimated cutoff scores generated by the faculty-derived modified Angoff method, the LLM-derived modified Angoff method, and the Cohen method were converted into operational cutoff scores by subtracting 1 SE of measurement. The SE of measurement was calculated according to classic test theory as follows:


$$SEM=SD\times \sqrt{1-r}$$

In this equation, *SD* is the SD of students’ raw examination scores, and *r* is the examination reliability (Cronbach α). The adjusted operational cutoff scores were subsequently used to determine students’ pass and fail classifications as this approach accounts for measurement error in observed examination scores and improves the defensibility of pass and fail decisions [21].

Paired-sample *t* tests were used to compare the mean estimates between the faculty judges and LLMs for the same examination items. A 2-way random-effects ANOVA, consistent with a generalizability (*G*) theory framework, was conducted separately for faculty judges and LLMs using a fully crossed item × rater design [22]. Variance components were estimated for items, raters, and the item × rater interaction, and the percentage of the total variance attributable to each component was calculated. Generalizability (*G*) coefficients were calculated to evaluate relative reliability, whereas Φ coefficients were calculated to evaluate absolute dependability for criterion-referenced decisions. A decision study estimated the projected *G* coefficients for panels ranging from 4 to 20 raters. In addition, the root mean squared error (RMSE) was calculated as a summary measure of the precision of the estimated Angoff cutoff scores, with lower values indicating greater consistency among raters. To calculate the RMSE, the following previously published formula was applied [18,23]:


$$\sqrt{\left({\hat{\sigma }}_{r}^{2}/n_{r}\right)+\left({\hat{\sigma }}_{ir}^{2}/(n_{i}n_{r})\right)}$$

In this equation, *n_i_* is the number of items, ${\hat{\sigma }}_{r}^{2}$ is the rater variance component, and ${\hat{\sigma }}_{ir}^{2}$ is the item × rater interaction variance component.

Pearson correlation was used to test the relationship between the mean value of item-related estimates for raters (faculty judges and LLMs) and the *P* value for each item (percentage of students answering each item correctly). The item *P* values were calculated from the same administration of the examination involving the 73 students included in this study. A *P* value below .05 was considered statistically significant. Data were analyzed using SPSS (version 24; IBM Corp). Generalizability theory analyses for standard setting estimates were performed using a publicly available web-based generalizability theory application (Reliability Angoff Calculator; shinyapps) [21].

## Results

### Overview

Seventy-three students completed the written examination. The mean raw examination score was 74.6% (SD 15.0%), with scores ranging from 38.3% to 100%. The examination demonstrated strong internal consistency (Cronbach α=0.91). The mean item discrimination was 0.32 (SD 0.12) with no negatively discriminating items. Table 1 shows the mean Angoff estimate scores and SDs from the 10 faculty judges and 5 LLMs. Faculty estimates ranged from a mean of 59.23 (SD 9.97) to 81.21 (SD 17.13). Among LLMs, DeepSeek produced the highest mean cutoff score (72.15, SD 20.06), whereas GPT-5.2 produced the lowest (66.18, SD 12.34).

**Table 1.** Mean Angoff estimates generated by faculty judges (n=10) and 5 large language models (GPT-5.2, Grok, DeepSeek, MedGemma, and Claude 4.5 Sonnet).

Rater type and model		Cut score (%), mean (SD)
Faculty judges		
	Judge 1	81.21 (17.13)
	Judge 2	68.62 (16.76)
	Judge 3	64.90 (16.72)
	Judge 4	63.11 (27.22)
	Judge 5	67.83 (15.17)
	Judge 6	63.96 (9.72)
	Judge 7	66.58 (22.42)
	Judge 8	59.23 (9.97)
	Judge 9	78.71 (8.00)
	Judge 10	77.17 (9.97)
LLMs		
	ChatGPT	66.18 (12.34)
	Grok	67.79 (14.29)
	DeepSeek	72.15 (20.06)
	Med-PaLM 2	71.88 (14.38)
	Claude Sonnet 4.5	66.68 (18.64)

### Differences Between the Angoff Estimates Generated by Faculty Judges and LLMs

A paired-sample *t* test showed that the Angoff cutoff scores produced by the LLMs (mean 68.94, SD 12.39) were closely comparable to those produced by faculty judges (mean 69.13, SD 10.92), and the difference between the 2 groups did not reach statistical significance (95% CI −2.21 to 2.91; *t*_119_=0.27; *P*=.79).

### LLM Conceptualization of the MCC and Estimation of Item-Level Probabilities of Percentage of Correct Responses

Table 2 shows that the LLMs differed in how they conceptualized the MCC and estimated the probability of a correct response. GPT-5.2, Claude 4.5 Sonnet, and Grok based their conceptualization on learner characteristics. GPT-5.2 and Claude 4.5 Sonnet described the MCC as a student with adequate basic knowledge who struggles with advanced mental tasks. Grok similarly emphasized predictable errors and challenges in distinguishing related concepts. DeepSeek used cohort GPA distribution to identify students near the pass threshold, whereas MedGemma focused on item characteristics rather than learner attributes.

LLMs also differed in estimating the percentage of correct responses. GPT-5.2 and Claude 4.5 Sonnet considered item-level features such as cognitive level, content familiarity, distractor quality, and likely misconceptions. DeepSeek and MedGemma focused on the complexity of the subject matter, the depth of conceptual knowledge required, and the cognitive processing required for answering each MCQ. Grok anticipated errors, misconceptions, and possible reasoning failures of students.

**Table 2.** Large language model (LLM) conceptualization of the minimally competent candidate (MCC) and estimation of item-level probability of correct response across LLMs.

LLMs	Conceptualization of the MCC	Process for estimating the percentage of correct responses
GPT-5.2	The MCC is a learner with adequate core knowledge but limited ability to apply higher-order reasoning, particularly in tasks requiring integration or multiple steps.	Estimates are derived through structured consideration of item characteristics, including cognitive level, familiarity, clarity, and the effectiveness of distractors. These elements are weighed to reflect the overall cognitive demand of the question.
Claude 4.5 Sonnet	The MCC is a learner with variable performance capable of handling straightforward items but less reliable when confronted with complexity or layered reasoning.	Estimates are generated using a comparative, justification-oriented process. Items are evaluated in relation to one another, with adjustments made to maintain consistency across the assessment rather than treating each item independently.
Grok	The MCC is characterized by a tendency toward common and predictable errors, such as misinterpretation of questions, confusion between similar concepts, or computational mistakes.	Estimates are informed by anticipated error patterns. The likelihood of incorrect reasoning pathways is considered when determining how often a borderline candidate would answer correctly.
DeepSeek	The MCC is defined operationally, with borderline candidates identified through GPA^a^ ranges approximating the pass threshold.	Estimates are determined through a stepwise analytical approach involving content review, difficulty classification, and justification of each estimate. Item-level probabilities are subsequently aggregated to inform the overall cutoff score.
MedGemma	The MCC is not explicitly described in terms of learner attributes; instead, performance is inferred relative to item difficulty.	A difficulty-based model is used in which the probability of a correct response decreases progressively with increasing item difficulty. This pattern is supported by both item-specific reasoning and overall trends.

a GPA: grade point average.

### Reliability of Angoff Estimates Among Faculty Judges Compared With LLMs

### Generalizability Theory Analysis

**Figure 1.**
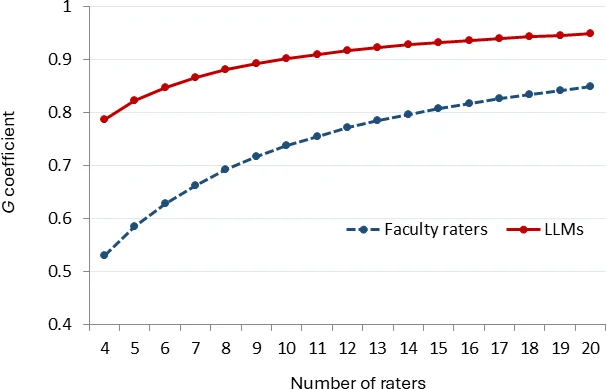
Decision study showing the effect of increasing the number of raters on the generalizability (*G*) coefficient of the modified Angoff procedure. The graph compares projected *G* coefficients for faculty judges and large language models (LLMs) across panel sizes ranging from 4 to 20 raters.

Table 3 and Table 4 summarize the generalizability theory analysis of the modified Angoff procedure for faculty judges and LLMs. Generalizability theory analysis demonstrated moderate reliability of the modified Angoff cutoff scores for the 10-member faculty panel. The generalizability (*G*) coefficient was 0.738, and the corresponding Φ coefficient was 0.692, indicating acceptable dependability for both relative and absolute pass and fail decisions. The decision study (Figure 1) demonstrated that reliability improved progressively as the number of faculty judges increased, with the *G* coefficient increasing from 0.530 with 4 judges to 0.849 with 20 judges.

**Table 3.** Generalizability theory analysis of Angoff estimates generated by faculty judges (n=10) and 5 large language models (LLMs; GPT-5.2, Grok, DeepSeek, MedGemma, and Claude 4.5 Sonnet): variance components.

Source of variance	Faculty judges (n=10)		LLMs (n=5)	
	Variance component (% points)²	Percentage of variance	Variance component (% points)²	Percentage of variance
Items	58.94	18.36	126.36	46.85
Raters	52.64	16.40	7.12	2.64
Item × rater	209.50	65.25	136.24	50.51
Total variance	321.08	100	269.72	100

**Table 4.** Reliability and precision indexes.^a^

Reliability and precision indexes	Faculty judges (n=10)	LLMs^b^ (n=5)
*G* coefficient^c^	0.738	0.823
Φ coefficient	0.692	0.815
RMSE^d^	2.33	1.28

a The *G* coefficient estimates relative reliability, whereas the Φ coefficient estimates absolute reliability for criterion-referenced decisions.

b LLM: large language model.

c Generalizability coefficient.

d RMSE: root mean squared error of the estimated Angoff cutoff scores.

Compared with the faculty panel, the 5 LLMs demonstrated greater psychometric consistency. The LLM panel achieved a *G* coefficient of 0.823 and a Φ coefficient of 0.815 despite comprising only 5 models. The decision study similarly showed progressive improvements in reliability with an increasing number of LLM raters, with the *G* coefficient increasing from 0.788 with 4 LLMs to 0.949 with 20 LLMs.

The generalizability theory analysis found that LLMs had much larger item-related variance than faculty judges (46.85% vs 18.36%), indicating that LLMs discriminated more between examination items. In contrast, rater-related variance was substantially smaller for LLMs than for faculty judges (2.64% vs 16.40%), indicating more consistency in LLM-generated Angoff estimations. The item × rater interaction was the most significant source of variance in both categories, accounting for 65.25% of the total variance among faculty judges and 50.51% among LLMs. Similarly, LLMs had a smaller RMSE than faculty judges (1.28 vs 2.33), indicating improved accuracy in the predicted cutoff scores.

### Failure Rates After Applying 3 Different Standards

The adjusted cutoff scores were 64.67% for the faculty-derived modified Angoff method, 64.48% for the LLM-derived modified Angoff method, and 68.20% for the Cohen method (Table 5). Applying these cutoff scores to students’ raw examination scores yielded similar pass and fail classifications for the faculty- and LLM-derived Angoff methods, with 71.2% (52/73) passing and 28.8% (21/73) failing. In contrast, the Cohen method produced a higher operational cutoff score, classifying 64.4% (47/73) of the pupils as passing and 35.6% (26/73) as failing.

**Table 5.** Standard setting outcomes and individual-level agreement for the modified Angoff method (faculty judges and large language models [LLMs]) and the Cohen method: operational cutoff scores and pass and fail outcomes. Pass and fail classifications were determined using students’ raw examination scores.

Standard setting method	Adjusted cutoff score (%)	Pass rate (n=73), n (%)	Fail rate (n=73), n (%)
Angoff method—faculty	64.67	52 (71.2)	21 (28.8)
Angoff method—LLMs	64.48	52 (71.2)	21 (28.8)
Cohen method	68.20	47 (64.4)	26 (35.6)

Individual-level agreement analysis demonstrated perfect agreement between the faculty- and LLM-derived Angoff methods (100% agreement; Cohen κ=1.000; exact McNemar *P*>.99). Comparisons between the Cohen method and each modified Angoff approach showed 93.2% overall agreement, with almost perfect agreement (κ=0.844) and no statistically significant differences in pass and fail classifications (exact McNemar *P*=.06 for both comparisons; Table 6).

**Table 6.** Individual-level agreement between standard setting methods.^a^

Comparison	Overall agreement (%)	Cohen κ	Exact McNemar *P* value
Angoff method—faculty vs LLMs^b^	100	1.000	>.99
Angoff method—faculty vs Cohen method	93.2	0.844	.06
Angoff method—LLMs vs Cohen method	93.2	0.844	.06

a Agreement was assessed using the Cohen κ and the exact McNemar test. A nonsignificant McNemar test indicates no statistically significant difference in paired pass and fail classifications.

b LLM: large language model.

### Relationship Between Angoff Estimates and Item Difficulty

Pearson correlations showed statistically significant positive relationships between Angoff estimates and observed item difficulty for both groups. The correlation was moderate for LLMs (*r*=0.552; *P*<.001) and weaker for faculty judges (*r*=0.437; *P*<.001). Because the difference between these dependent correlations was not formally tested, these coefficients are presented descriptively rather than as evidence that one correlation was statistically greater than the other.

## Discussion

### Principal Findings

The Angoff method is the most commonly used standard setting approach in health professions education [1,20,24]. This is, to our knowledge, the first published study comparing LLMs and human judges in Angoff standard setting for medical education. Four key findings emerged. First, paired analysis showed that Angoff estimates from 10 faculty judges and 5 LLMs did not differ significantly, resulting in comparable operational cutoff scores and identical pass and fail classifications. Second, generalizability theory analysis found acceptable reliability for both the faculty and LLM panels, with reliability increasing as the number of raters grew. The LLM panel also showed lower rater-related variation, lower RMSE, and higher item-related variance than the faculty panel, indicating better consistency and discrimination across the evaluated LLM outputs. Third, there was significant heterogeneity in how LLMs conceptualized the MCC and estimated item levels. Fourth, both faculty- and LLM-derived Angoff estimates showed moderate positive relationships with reported item difficulty, supporting, but not conclusively, criterion-related validity. Overall, these data indicate that LLMs may be helpful as adjuncts to faculty judges in modified Angoff standard setting while emphasizing the importance of expert human judgment in high-stakes assessment.

### Implications of the Findings

The lack of significant differences between LLM and faculty estimates suggests that, with appropriate contextual information and structured prompts, LLMs can approximate expert human judgment in predicting whether a minimally competent student will answer an item correctly. This is important because the credibility of the Angoff method depends on the quality of judges, their conceptualization of the MCC, and their accuracy in predicting item scores [23]. Credibility in standard setting is defined by stakeholders’ trust that the cutoff scores are appropriate, defensible, and fit for purpose, best demonstrated through a validity argument framework using multiple sources of evidence [25].

This study provides 3 sources of validity evidence. The first is *procedural validity*. The faculty-based standard-setting process involved an adequate number of trained judges, a clear MCC definition, explicit guidance on the modified Angoff method, and access to historical student performance data. The same information and standardized prompts were given to each LLM, ensuring consistent instructions for all AI raters.

The second source is *internal validity*, reflecting the degree of stability of Angoff estimates in response to sources of measurement errors such as judges or items [18]. Generalizability theory was used instead of a conventional interrater agreement coefficient because it partitions measurement error into components attributable to items, raters, and their interaction [22]. The faculty panel achieved *G* and Φ coefficients that demonstrated moderate reliability for relative and absolute pass and fail decisions. On the other hand, the 5-LLM panel demonstrated higher reliability despite comprising only half as many raters as the faculty judges. Consistent with these findings, the RMSE was lower for LLMs than for faculty judges, indicating greater consistency of the evaluated LLM outputs under the standardized prompting conditions.

Examination of the variance components provides additional insights into these findings. The rater-related variance among LLMs was 6 times lower than that among faculty judges, which suggests greater agreement under standardized prompting conditions. Previous studies using progress tests have reported even lower rater-related variance, likely reflecting differences in assessment context, examination format, and judge characteristics rather than differences in the standard setting method itself [18,23]. In contrast, item-related variance for LLMs was 2.5 times higher than that for faculty judges. These findings suggest that Angoff estimates for LLMs were influenced more by differences in item characteristics, whereas faculty judgments were affected to a greater extent by rater-specific variation. Nevertheless, the substantial item × rater interaction in both groups indicates that variability in ratings remained largely dependent on individual examination items. However, the greater consistency observed among LLMs should not be interpreted as evidence of superior validity. The convergence among LLMs may partly reflect similarities in their training data, model architectures, and response patterns rather than fully independent judgments. Therefore, although the findings support the reliability of LLM-assisted standard setting, human expert judgment remains essential for evaluating content relevance, contextual appropriateness, and the expected performance of an MCC.

The third source of evidence is *external validity*, assessed in two ways. First, faculty- and LLM-derived cutoff scores produced similar pass rates, indicating comparable practical outcomes for students. The Cohen norm-referenced method resulted in a much higher failure rate, consistent with existing literature [20]. Second, LLM Angoff estimates demonstrated a moderately stronger correlation with observed item difficulty than faculty estimates, indicating closer alignment with observed difficulty of test items. However, this stronger correlation should be interpreted cautiously as item *P* values reflected the overall cohort performance rather than that of MCCs. Therefore, these findings provide supportive rather than definitive evidence of external validity. Collectively, these findings support LLMs as credible contributors to modified Angoff standard setting while highlighting the ongoing need for informed human decision-making in high-stakes assessment.

The SAMR model provides a useful conceptual lens for interpreting the potential role of LLMs in modified Angoff standard setting [16]. On the basis of the observed findings, LLMs appear to align with the substitution and augmentation levels of the framework, whereas we recognize that this interpretation is conceptual rather than an empirical outcome of the present study. At the substitution level, LLMs replicated the core faculty role by conceptualizing the MCC and generating Angoff estimates comparable to those of expert judges. Within the SAMR framework, these findings may be interpreted as aligning with the augmentation level because the evaluated LLMs demonstrated greater consistency under standardized prompting conditions. The diverse reasoning approaches among LLMs, from cognitive feature–based and difficulty-driven models to error anticipation, highlight their potential as cognitive partners in assessment decision-making [26]. These findings support a practical role for LLMs as substitutes for or augmentations to the human Angoff process. LLMs could improve efficiency, reduce faculty cognitive burden, and enhance the reliability of standard setting while maintaining necessary human oversight and organizational accountability in high-stakes assessment. Future research should examine whether more advanced applications of LLMs support progression to the modification or redefinition levels of the SAMR model.

### Limitations

Several limitations should be acknowledged. This was a single-institution study with one 120-item fundamental sciences exam, 10 faculty judges, and 5 selected LLMs, which may not reflect the broader and quickly changing environment of LLMs, limiting generalizability. Each LLM was evaluated in a single standardized run; therefore, within-model stochastic variability and reproducibility across multiple sessions were not assessed. As a result, the apparent consistency across the analyzed LLMs may be due to commonalities in their underlying models rather than independent expert-like assessments. Standardized prompting protocols do not guarantee consistent results across different LLMs or future model versions. Another limitation is that both faculty judges and LLMs were given the same historical cohort GPA data before estimating item performance. While this reflects recognized Angoff practice, the common performance anchor may have contributed to the convergence of faculty and LLM estimations, and future research should compare LLM performance with and without such contextual information. Although all judges were given standardized Angoff instructions, variances in their core disciplinary competence may have contributed to some interrater variation. Finally, this study focused only on type-A MCQs, so findings may not apply to other assessment formats, such as objective structured clinical examinations. Future research should seek to address these limitations by examining reproducibility across multiple institutions, disciplines, assessment formats, and standard setting methods. Longitudinal studies that monitor the influence of AI-assisted standard setting on student achievements would be extremely beneficial.

### Conclusions

In this single-institution study, LLM-generated Angoff estimates were broadly comparable to faculty-generated estimates, with better generalizability coefficients and more consistency across the tested outputs under uniform prompting settings. These findings lend support to the prospective use of LLMs as adjuncts to faculty in standard setting. However, they do not show that LLMs are equivalent to or superior to expert judges, nor do they support their independent use in high-stakes assessments. However, more multicenter studies with multiple assessment formats, repeated model evaluations, and a broader range of LLMs are required to determine the generalizability and reproducibility of these findings.

## Supplementary material

10.2196/98766 Multimedia Appendix 1 Prompts used for the large language models in this study.

## Abbreviations

GPA: grade point average
LLM: large language model
MCC: minimally competent candidate
MCQ: multiple-choice question
RMSE: root mean squared error
SAMR: substitution, augmentation, modification, and redefinition
