# Supplementary material for: AI-Assisted Angoff Standard Setting for Multiple-Choice Examinations in Medical Education: Comparative Study
Source: JMIR Med Educ. 2026 Sep 9;12:e98766. doi: 10.2196/98766 (PMC13557613; doi:10.2196/98766)
Supplement: Supplementary file 1 [file mededu-v12-e98766-s001.pdf]

Appendix 1: *Prompts used for the Large Language Models in the study*

1) *Prompts used to identify the expected percentage of MCC students who can score each item correctly.*

Prompt 1:

Role:

Medical Educator with expertise in assessment and standard setting by Angoff method.

Task:

I will give you the grade book of a cohort and a question paper. Based on cumulative GPA you will decide the borderline group and then for each MCQ you will tell me the percentage of borderline students that will answer it correctly and your rationale justifying why you gave that percentage. Are you ready?

Prompt 2:

Cumulative GPA with anonymized study identification code (file attached)

Prompt 3:

Analyse the 120 MCQ's in the file and give me the answer as a Table with 4 columns, Serial number, MCQ (Paste the MCQ here), Difficulty level, and Angoff predicted percentage. (Question paper file attached)

2) Prompts aiming to test how each large language model defined the minimally competent candidate and how they came up with the percent of students who can answer each item correctly.

Role:

You are a medical educator with expertise in assessment and standard setting using the Angoff method.

Task:

You have estimated the percentage of borderline (minimally competent) students expected to answer each MCQ correctly. Now, explain the reasoning framework you used to arrive at these percentage estimates.

Specifically describe:

1. How you conceptualized a borderline (minimally competent) student
2. What factors you considered when estimating the percentage for each item (e.g., cognitive level, content familiarity, clarity of the question, distractor quality)
3. How you differentiated between easy, medium, and hard questions
4. How you determined approximate percentage ranges (e.g., 70–85%, 50–70%, 30–50%)
5. How you handled uncertainty or ambiguity in the questions

Output Format:

Provide a structured explanation under the heading:

"AI Explainability of Angoff Estimation"

3)Purpose of the Prompt Framework

This framework integrates Angoff standard setting with AI explainability, enabling simultaneous evaluation of both predicted performance and underlying reasoning processes. It supports transparency, reproducibility, and systematic analysis of AI-assisted decision-making in assessment.

4. Reproducibility Statement

All prompts were applied in a standardized manner without iterative human correction unless explicitly specified.
